# Does the sizing of current cervical disc arthroplasty systems match Chinese cervical anatomic dimensions?

**DOI:** 10.3389/fbioe.2022.1036223

**Published:** 2022-10-25

**Authors:** Lu Wang, Meng Bai, Xing-Bin Li, Zhao-Rui Wang, Bang Wang, Ai-Bing Huang

**Affiliations:** ^1^ Postgraduate School, Dalian Medical University, Dalian, Liaoning, China; ^2^ Department of Orthopedics, Taizhou People’s Hospital Affiliated to Nanjing Medical University, Taizhou, Jiangsu, China; ^3^ Department of Orthopedics, Xi’an People’s Hospital, Xi’an, Shaanxi, China

**Keywords:** anatomical measurement, cervical disc replacement, prosthesis, match, cervical disc arthroplasty

## Abstract

**Objective:** The objectives of this study were to analyze the computed tomography (CT) scan imaging data of the cervical spine from healthy volunteers and to correlate the measurements to the dimensions of current cervical disc arthroplasty systems.

**Methods:** A total of 130 participants (78 males and 52 females) with a mean age of 41.0 years (range 18.0–66.0 years) who had undergone computed tomography scans of the cervical spine were included. The linear parameters of the C3 to C7 levels, including anterior-posterior diameter (AP), middle disc height (DH), anterior disc height (ADH), posterior disc height (PDH) and center mediolateral diameter (ML), were measured. The analysis was conducted comparing different cervical levels, sexes, and age groups. Known dimensions from eight cervical disc arthroplasty systems were compared with the morphologic data.

**Results:** A total of 520 vertebral segments were measured. The mean values for the measured parameters were as follows: anterior-posterior diameter 16.08 ± 1.84 mm, mediolateral diameter 16.13 ± 1.99 mm, anterior disc height 3.88 ± 1.11 mm, disc height 5.73 ± 1.00 mm, posterior disc height 2.83 ± 0.94 mm, and mediolateral diameter/anterior-posterior diameter 1.01 ± 0.13. All parameters except for posterior disc height were significantly different across the different cervical levels (*p* < 0.05). There were also significant sex differences in terms of the linear parameters. No differences were found in the majority of parameters among the different age groups (*p* > 0.05), except for anterior-posterior diameter at the C6/7 level. A comparison of the bone dimensions from the study data and the dimensions of the implants indicated the presence of a size mismatch in the currently available cervical disc prostheses.

**Conclusion:** There is a large discrepancy between the cervical anatomical data of Chinese patients and the sizes of currently available prostheses. The dimensions collected in this study could be used to design and develop appropriate disc prostheses for Chinese patients.

## Introduction

Cervical spondylosis is a degenerative condition of the intervertebral discs and vertebral bodies, in which compression of the cervical nerve root or spinal cord leads to several motor and sensory dysfunctions. Essential surgical treatments may be indicated for patients with persistent radicular pain after conservative treatment and profound or progressive motor weakness. Anterior cervical decompression and fusion (ACDF) was first described by Smith and Robinson in the 1950s, and since then, it has been widely performed for the treatment of degenerative disc disease associated with radiculopathy or myelopathy (Smith and Robinson, 1958). However, ACDF sacrifices segmental mobility, and the fusion of one or more segments in this procedure may increase stress and motion at the adjacent unfused segments, accelerating adjacent segment degeneration (ASD) and spondylotic changes (Donk et al., 2018; Vleggeert-Lankamp et al., 2019).

Over the past several years, cervical disc replacement (CDR) has been extensively introduced to restore mobility at the operated segment and decrease the aforementioned stress, which in turn should prevent the development of ASD due to ACDF (4). Despite the good prospects for this technique, some adverse complications have been reported, namely, heterotopic ossification (HO), prosthesis migration and subsidence, bone loss, and segmental kyphosis (Li et al., 2019; Virk et al., 2021). While the reasons for the development of these conditions are undoubtedly complex, some reports have revealed that many of these factors are associated with the mismatch between the dimensions of the cervical endplates and the footprints of the prostheses (Thaler et al., 2013; Guo et al., 2020). Although differences in cervical endplate size among different races was previously confirmed (Yao et al., 2018), few artificial discs are designed specifically to satisfy the demand of the Chinese population. At present, an increasing number of types of artificial cervical disc prostheses have received approval from the U.S. Food and Drug Administration (FDA). Regionally, do the sizes of current prostheses exactly meet the anatomical characteristics of the cervical spine in East Chinese adults? Supportive anthropometric evidence for answering this question is lacking.

Consequently, the purpose of the present study was to compare the parameters of cervical vertebrae in the Chinese population with the sizes of eight artificial cervical disc prostheses approved by the FDA and to provide reference data for the design of future cervical devices.

## Materials and methods

This is a retrospective study. After approval of our ethics committee, we collected data from 130 participants (78 males and 52 females) with a mean age of 41 years (range 18–66 years) who underwent CT scans of the cervical spine between January 2015 and December 2018. Age was categorized into three groups for analysis: Group A (<35 years), Group B (35–49 years), and Group C (≥50 years) (Table 1).

**TABLE 1:** Study population categorized by sex and age.

Groups	Age	n (130)	
		Male	Female
Group A	<35	24	16
Group B	35–49	31	18
Group C	≥50	23	18

All participants were recruited, and those without signs of anatomical anomalies and no obvious degenerative conditions of the cervical spine (e.g., cervical disc herniation, osteophyte formation and ossification of the anterior or posterior longitudinal ligament) in the CT examination were included in the study, while patients with congenital dysplasia, tumor, bone fracture, infection, and prior cervical spine surgery were excluded.

CT scans were performed for all patients with 64-slice multidetector row CT scanners. All images were transferred to a picture archiving and communication system (PACS) and were measured directly using the built-in tools of the PACS workstations.

**FIGURE 1:**
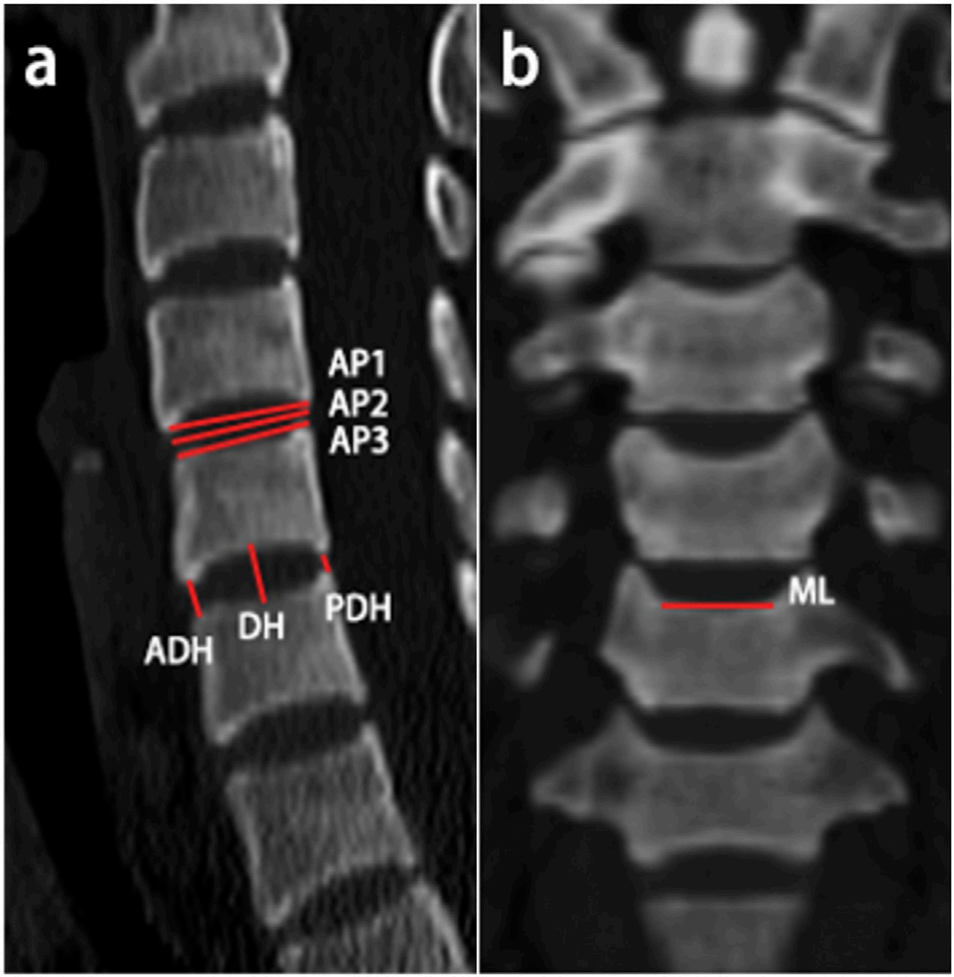
Schematic representation of the measured linear parameters. **(A)** The anterior‐posterior diameter and anterior/middle/posterior disc height were measured in sagittal CT scans; **(B)** The center mediolateral diameter of the superior vertebral endplates was measured in coronal CT scans.

For better determine the dimension, the measurement was taken from the mid-sagittal plane and the mid-coronal plane. The linear parameters of each intervertebral segment (C3/4, C4/5, C5/6, and C6/7) were measured as follows: (Food and Drug Administration, 2007a) the anterior-posterior (AP) diameter, measured as the mean value of the AP diameters of the superior endplate (AP1), middle intervertebral space (AP2) and inferior endplate (AP3) in sagittal CT scans to minimize measurement errors (Figure 1A); (Vleggeert-Lankamp et al., 2019) the disc height of the anterior (ADH), middle (DH) and posterior (PDH) disc space (Figure 1A); and (Donk et al., 2018) the center mediolateral (ML) diameter of the superior endplates in coronal CT scans (Figure 1B). The above measurements were taken by two of the authors. Mean data was obtained for final analysis.

**FIGURE 2:**
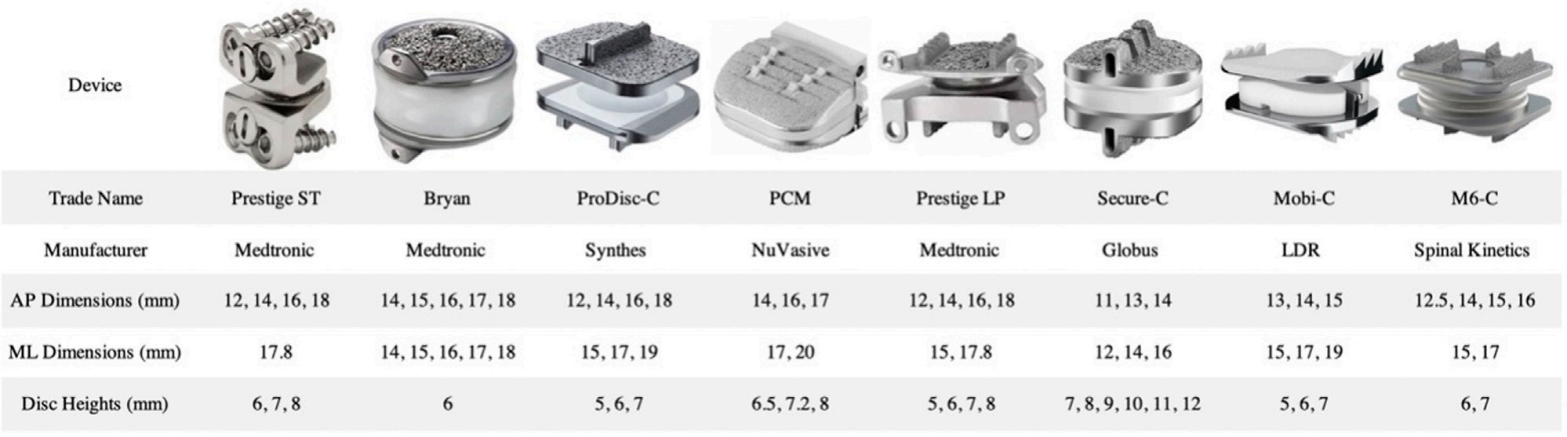
Prosthesis information: Prestige ST (10), Bryan (Food and Drug Administration, 2009), ProDisc-C (12), PCM(13), Prestige LP (14, 15), Secure-C (16), Mobi-C (17, 18), M6-C (19).

For the matching performance evaluation, cervical measurements were compared with the sizes of eight current, FDA-approved brands of artificial cervical disc prostheses: Prestige ST (Medtronic), Bryan (Medtronic), ProDisc-C (Synthes), PCM (NuVasive), Prestige LP (Medtronic), Secure-C (Globus), Mobi-C (LDR) and M6-C (Spinal Kinetics). The sizes of each prosthesis are listed in Figure 2).

Statistical analysis was performed using Excel (Microsoft Excel 2019), SPSS (IBM SPSS Statistics 26), and Prism software (GraphPad Prism 8). Based on Skewness and Kurtosis, the data showed normal distribution. The measurement data are expressed as the mean ± standard deviation (x¯ ± *s*). Differences between groups were compared by independent sample t tests or one-way analysis of variance. *p* values less than 0.05 (*p* < 0.05) were considered statistically significant.

## Results

A total of 130 patients with 520 vertebral segments were included in the analyses. The mean values for each measurement were as follows: AP 16.08 ± 1.84 mm, ML 16.13 ± 1.99 mm, ADH 3.88 ± 1.11 mm, DH 5.73 ± 1.00 mm, and PDH 2.83 ± 0.94 mm. Morphological CT scan data are presented in Table 2. All measurements except PDH were significantly different among cervical segments.

**TABLE 2:** Dimensions of Linear Parameters (‾X ± S, mm).

Dimensions	C3/4	C4/5	C5/6	C6/7	Total
AP	15.42 ± 1.64	15.78 ± 1.81	16.42 ± 1.88	16.72 ± 1.76	16.08 ± 1.84
ML	14.58 ± 1.45	15.45 ± 1.41	16.59 ± 1.59	17.91 ± 1.72	16.13 ± 1.99
ADH	3.37 ± 0.97	3.74 ± 1.03	3.74 ± 1.17	4.42 ± 1.00	3.88 ± 1.11
DH	5.51 ± 0.99	5.59 ± 0.92	5.59 ± 0.96	6.21 ± 0.99	5.73 ± 1.00
PDH	2.96 ± 0.92	2.94 ± 0.97	2.94 ± 0.94	2.72 ± 0.92	2.83 ± 0.94

The analysis results for males and females are shown in Table 3. The majority of comparisons were statistically significant (*p* < 0.05). Nevertheless, there was a marginally significant difference in the ML at the C4/C5 segment (15.63 ± 1.47 mm vs. 15.18 ± 1.28 mm, *p* = 0.07). There was no significant difference in ADH at either C3/4 (3.46 ± 1.02 mm vs. 3.22 ± 0.87 mm, P=0.18) or C5/6 [4.16 ± 1. (Food and Drug Administration, 2012a) mm vs. 3.76 ± 1.20 mm, P=0.06]. The DH at the C5/6 segment was 5.72 ± 1.04 mm in males and 5.44 ± 0.82 mm in females, showing no statistically significant difference (*p* = 0.10). The DH at the C6/7 segment was 6.31 ± 1.02 mm in males and 6.06 ± 0.92 mm in females, showing no statistically significant difference (*p* = 0.16). Additionally, there was no significant difference in the PDH at C4/5 (3.05 ± 1.01 mm vs. 2.76 ± 0.89 mm, *p* = 0.09), C5/6 (2.74 ± 0.97 mm vs. 2.66 ± 0.89 mm, *p* = 0.61) or C6/7 (2.62 ± 0.94 mm vs. 2.79 ± 0.91 mm, *p* = 0.30).

**TABLE 3:** Sex differences in Linear Parameters (‾X ± S).

Dimensions	Group	C3/4	C4/5	C5/6	C6/7	Total
AP (mm)	Male	16.29 ± 1.39	16.63 ± 1.57	17.27 ± 1.82	17.57 ± 1.59	16.94 ± 1.67
	Female	14.10 ± 0.98	14.49 ± 1.33	15.14 ± 1.11	15.44 ± 1.14	14.80 ± 1.25
	*p* value	0.00	0.00	0.00	0.00	0.00
ML (mm)	Male	14.92 ± 1.33	15.63 ± 1.47	16.90 ± 1.59	18.45 ± 1.62	16.47 ± 2.01
	Female	14.09 ± 1.49	15.18 ± 1.28	16.11 ± 1.50	17.12 ± 1.58	15.62 ± 1.84
	*p* value	0.00	0.07	0.01	0.00	0.00
ADH (mm)	Male	3.46 ± 1.02	3.98 ± 1.05	4.16 ± 1.13	4.64 ± 0.95	4.06 ± 1.12
	Female	3.22 ± 0.87	3.38 ± 0.89	3.76 ± 1.20	4.09 ± 0.98	3.61 ± 1.04
	*p* value	0.18	0.00	0.06	0.00	0.00
DH (mm)	Male	5.86 ± 0.94	5.91 ± 0.84	5.72 ± 1.04	6.31 ± 1.02	5.95 ± 0.98
	Female	4.98 ± 0.82	5.13 ± 0.84	5.44 ± 0.82	6.06 ± 0.92	5.4 ± 0.94
	*p* value	0.00	0.00	0.10	0.16	0.00
PDH (mm)	Male	3.16 ± 0.96	3.05 ± 1.01	2.74 ± 0.97	2.62 ± 0.94	2.94 ± 0.97
	Female	2.67 ± 0.79	2.76 ± 0.89	2.66 ± 0.89	2.79 ± 0.91	2.68 ± 0.87
	*p* value	0.00	0.09	0.61	0.30	0.00

**FIGURE 3:**
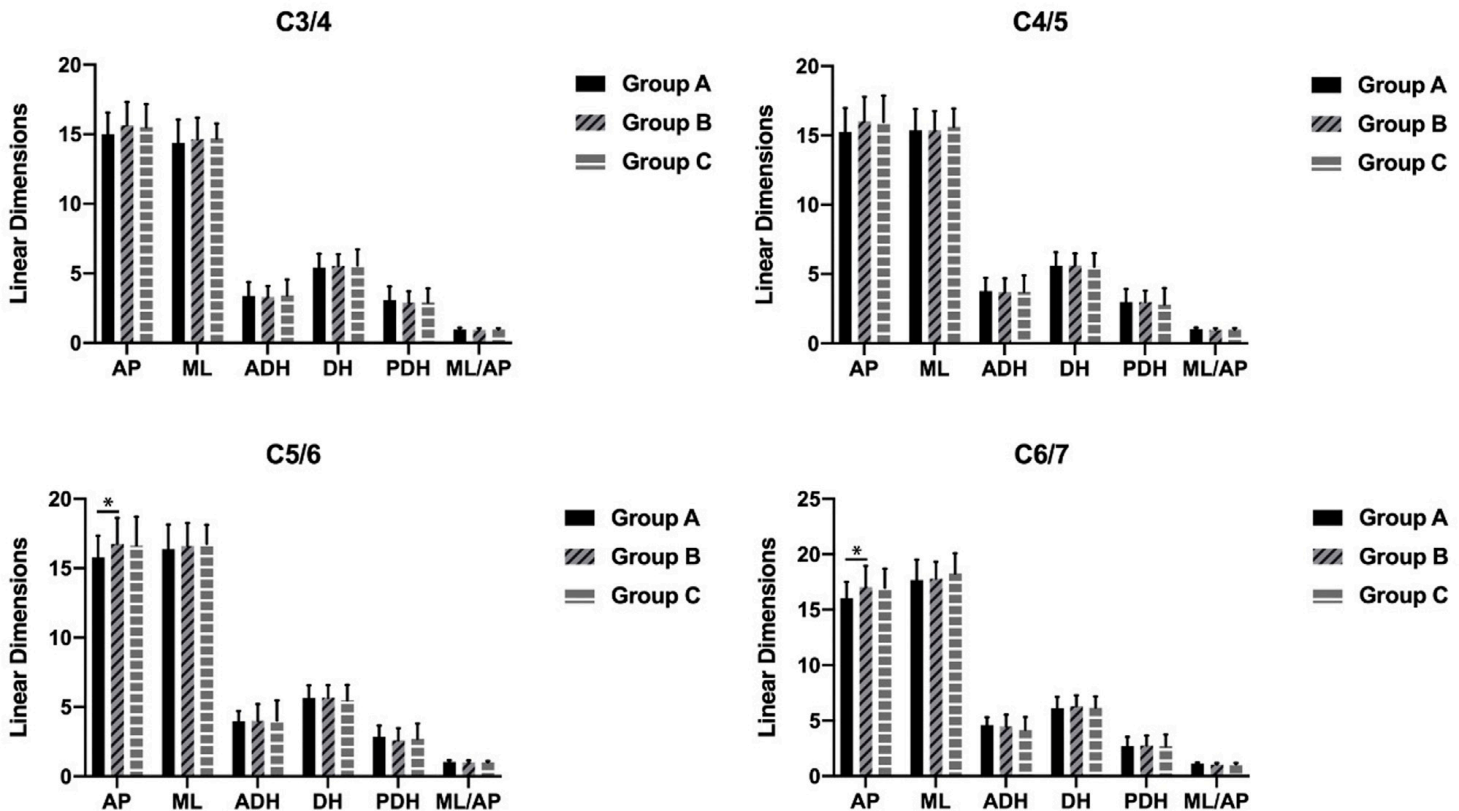
Differences among different age groups (*p < 0.05).

The AP in group B was significantly larger than that in group A at the C5/C6 and C6/C7 segments (*p* = 0.04 and *p* = 0.02). There were no other differences in the parameters between the age groups (Figure 3).

**FIGURE 4:**
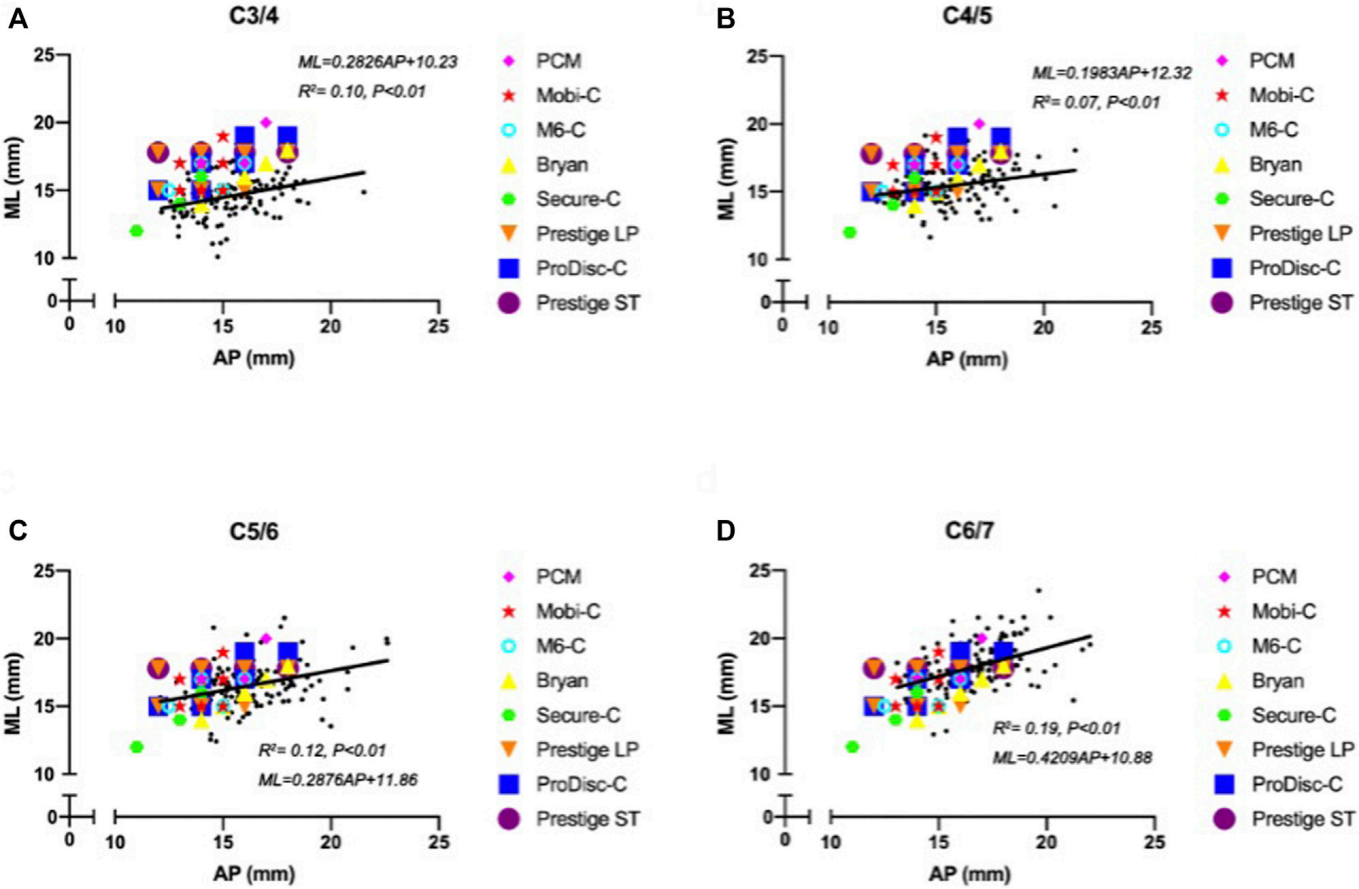
Mismatch between cervical anatomical data and the sizes of prostheses.

The width of the ML increased with increasing AP. Figure 4 shows the surprising differences between the footprints of the cervical disc prostheses and the cervical measurement distribution. A notable proportion of mediolateral prosthesis diameters were larger than the ML when the anterior-posterior diameters of the prostheses matched the AP distribution at the C3/4 segment (Figure 4A). At the C4/5 segment, the matching degree between the mediolateral diameters of the prostheses and the ML was better than that at the C3/4 segment, but the mediolateral diameters of the prostheses were mismatched for smaller values of the ML. The matching degree between the anterior-posterior diameters of the prostheses and the AP was lower than that at the C3/4 segment, and the anterior-posterior diameters of the prostheses were mismatched for larger values of the AP (Figure 4B). The highest matching degree between the mediolateral diameters of the prostheses and the ML was observed at the C5/6 segment, but the anterior-posterior diameters of the prostheses were mismatched for large values of the AP (Figure 4C). At the C6/7 segment, all implant devices were too small to match the measured ML and AP values (Figure 4D). In general, the matching degree between the AP and ML and the sizes of the different types of prostheses were unsatisfactory.

**FIGURE 5:**
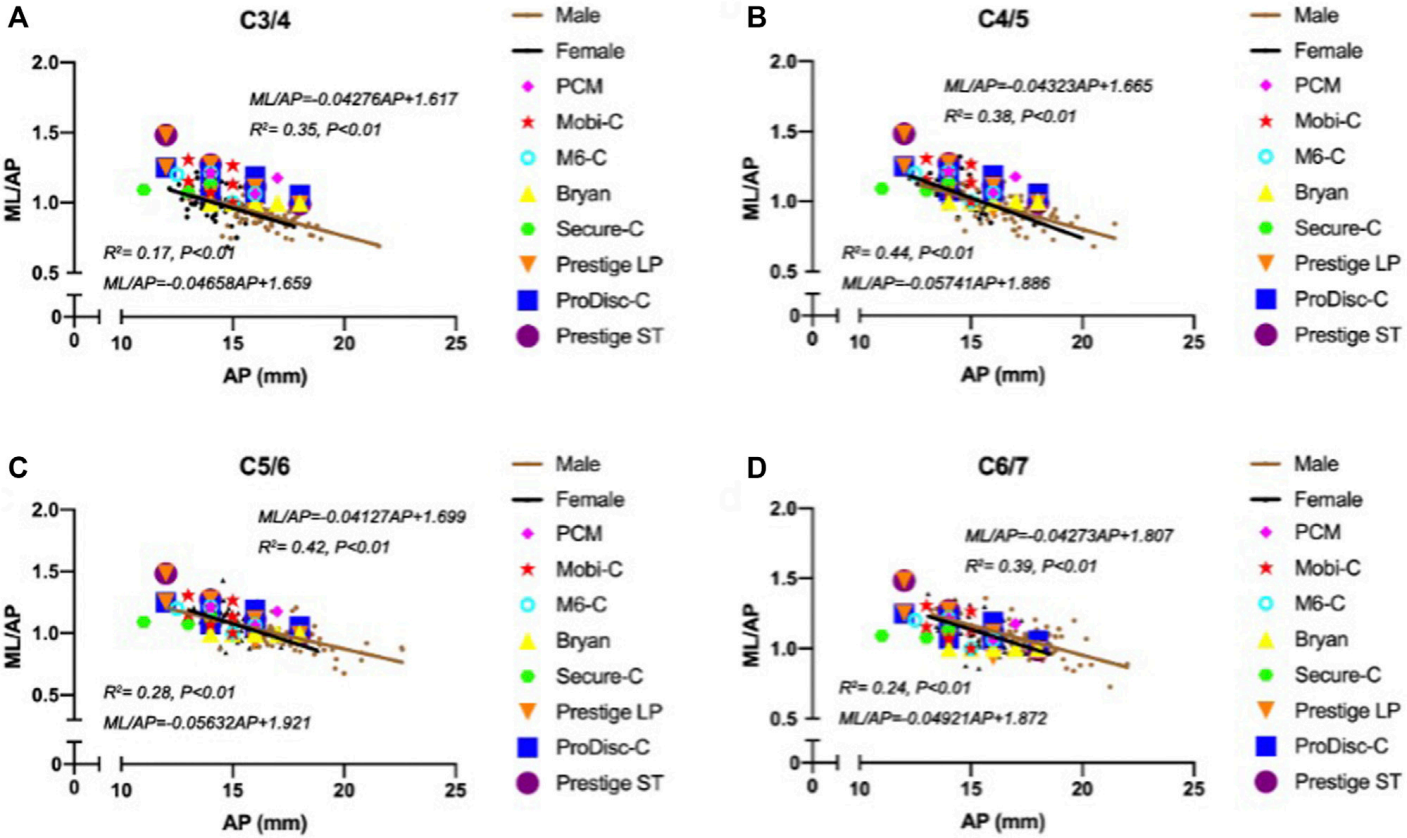
Mismatch of the width-to-depth ratio between cervical anatomical data and prostheses.

Moreover, a negative correlation was observed between ML/AP and AP, and the slopes for women were steeper than those for men with increasing AP between cervical segments. Again, the width-to-depth ratio of most designs, which represents the degree of endplate asymmetry, did not follow similar trends well, particularly for the Bryan device (Figure 5).

**FIGURE 6:**
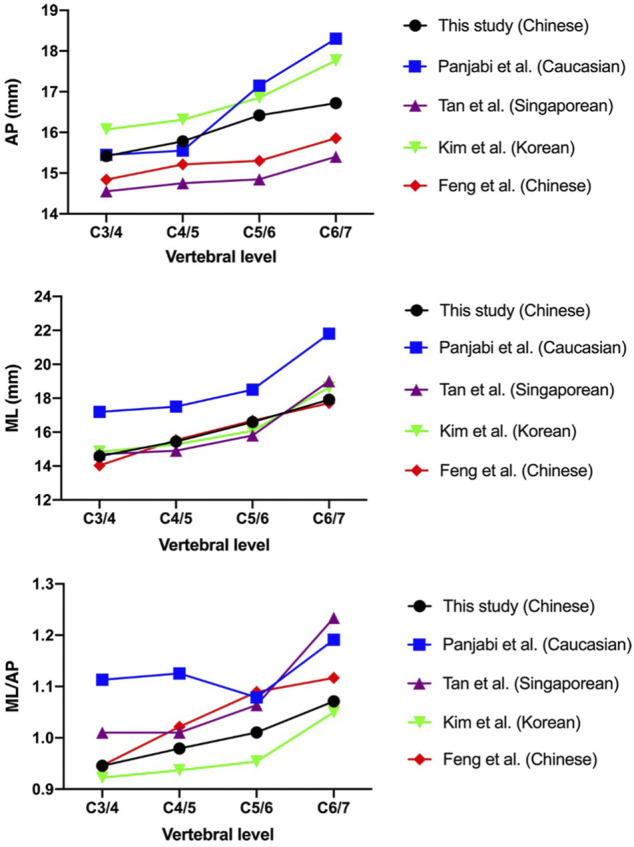
Comparison of the measurements of the present study with those reported by previous studies.

Many studies have reported on the measurement of cervical spine endplates (Kim et al., 1976; Panjabi et al., 1976; Tan et al., 2004; Feng et al., 2017). The results in the present study are shown compared with previous measurements from different parts of the world in Figure 6.

**FIGURE 7:**
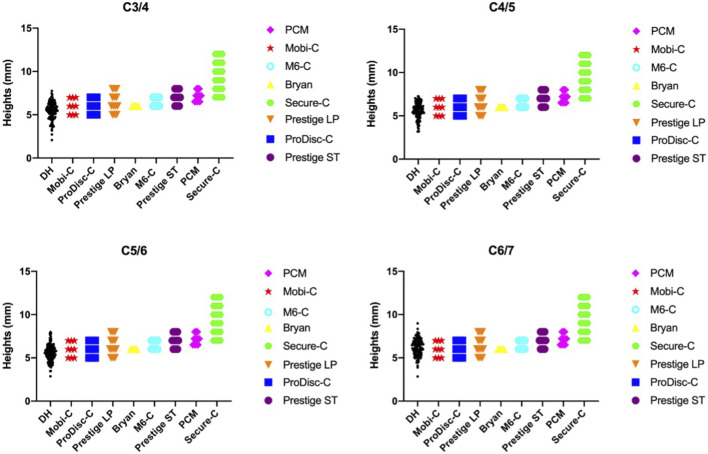
Mismatch between cervical anatomical data and the sizes of prostheses.

The minimum height of the prostheses was 5 mm (Mobi-C, ProDisc-C, Prestige LP), and the maximum prosthetic height was (Food and Drug Administration, 2007b) mm (Secure-C). The DH measurements ranged from 2.07 to 8.98 mm among the 130 patients. A considerable proportion of DH measurements were smaller than the minimum height of the available prostheses, as shown in Figure 7. Up to 34 patients had smaller DH values at C3/4 (26.15%), 31 patients had smaller values at C4/5 (23.85%), 33 patients had smaller values at C5/6 (25.38%) and 13 patients had smaller values at C6/7 (10%).

## Discussion

Over the past few decades, ACDF has become the standard procedure for the effective treatment of degenerative cervical spondylosis. Excessive compensatory activity, however, may lead to ASD, and some complications, including bone graft nonunion, implant migration, subsidence and bone donor site pain, have been observed during long-term follow-up (Yan et al., 2017). In contrast, as a nonfusion decompression method, CDR has become a more favored strategy in terms of preserving the motion of index segments and natural cervical kinematics (Findlay et al., 2018). Many biomechanical and clinical studies have shown that as an alternative to ACDF, CDR not only achieves similar outcomes but also reduces the incidence of ASD through motion preservation (Zou et al., 2017; Zhao and Yuan, 2019). In addition, CDR has been undergoing constant improvements and evidence-based redesigns to reduce prosthesis-related complications, such as implant dislocation, subsidence, migration, device wear and HO, which are often-mentioned side-effects (Lin et al., 1976), although the majority of them are usually asymptomatic in the short term and can be mitigated to a certain degree by proper patient selection and attention to the surgical technique (Salari and McAfee, 2012).

In our study, there was a large discrepancy between the cervical anatomical data of Chinese individuals and the footprints of currently available prostheses. The footprint is the part of the disk prosthesis designed to cover the endplate of the vertebra. Size matching between the prosthesis and cervical vertebra can not only provide a greater contact area between the prosthesis and cervical endplate but can also cover the peripheral marginal zones of the cervical endplate, which provides much stronger support than the central areas. Our results are consistent with other studies demonstrating the presence and prevalence of serious footprint mismatch. Thaler et al. (Thaler et al., 2013) measured the dimensions of cervical vertebrae from the CT scans of 24 patients and assessed the accuracy of matches achieved with common cervical disc prostheses (Bryan, Prestige LP, Discover, ProDisc-C). Overall, they found that compared with the cervical endplate diameters, 53.5% of the largest device footprints were smaller in their anterior-posterior diameter, and 51.1% were smaller in the mediolateral diameter. In the same manner, Dong et al. (Dong et al., 2015) reported that the mismatch in the available dimensions of prostheses and the anatomic data of cervical endplates ranged from 17.03% to 57.61% in the anterior‐posterior diameter and 35.51%–94.93% in the mediolateral diameter. In fact, given that there is a one-to-one correspondence between the measurements of cervical vertebrae and the sizes of cervical disc prostheses in a practical clinical setting rather than a size comparison of the frequency distribution, the true mismatching degree of prostheses might have been underestimated.

Footprint mismatch has been implicated as a major contributor to the development of prosthesis-related complications such as implant subsidence, migration and HO[(Food and Drug Administration, 2019), (Tu et al., 2012)]. An oversized implant may protrude and thus compress the nerves and soft tissues, which can further cause clinical issues and diseases. Undersized footprints could potentially cause subsidence and dislocation because of inadequate load distribution (Lin et al., 1976; Thaler et al., 2013). Guo et al. (Guo et al., 2020) reported that the mean footprint matching degree was 0.877 ± 0.068 in the sagittal plane and 0.852 ± 0.092 in the coronal plane. The mean overall footprint matching degree was 0.699 ± 0.102, and HO occurrence was significantly related to footprint mismatch. In multivariable analysis, Yang et al. (Yang et al., 2019) showed that patients with residual exposed endplates larger than 2 mm 4.5 times more likely to develop high-grade HO (*p* = 0.02) than patients with residual exposed endplates less than or equal to 2 mm. Therefore, maximizing the implant-endplate interface may help to reduce high-grade HO and preserve motion. Following data analysis, the ML/AP among different segments and sexes showed a statistically significant difference in our study. From the C3/4 to C6/7 discs, the vertebral endplate gradually becomes more elliptical (Feng et al., 2017). Although many prosthesis models are currently available for CDR, our findings indicated that the width-to-depth ratio of most designs did not follow similar trends well. Considering the above, it is important to design an artificial disc that imitates the shape of the endplates adjacent to a natural disc in all three dimensions.

The height of the artificial cervical disc prosthesis is mainly designed according to the middle disc height. Our study found that a considerable proportion of disc height measurements were less than the minimums height of available implants. An appropriate artificial disc height can achieve near-normal biomechanical properties. The increased disc height could result in decreased overlap of the facet joint articulation, reducing the restriction of flexion-extension motion, which would facilitate cervical rotation in the sagittal plane. A few studies (Peng et al., 2009; Kang et al., 2010) have suggested that the postoperative intersegmental range of motion is affected by disc height or disc height increment. Prostheses with heights ≥2 mm greater than normal can lead to marked changes in the abovementioned cervical biomechanics and bone-implant interface stress, which may induce ASD and subsidence (Yuan et al., 2018). Thus, when selecting an appropriate cervical implant, surgeons should consider patient height as well as estimated normal disc height.

Possible reasons for footprint mismatch include the following: first, the footprint dimensions of currently available disk prostheses were derived from early white cadaver data, and anatomic studies illustrated a large discrepancy between the footprint dimensions and anatomic data (Thaler et al., 2013; Karaca et al., 2016). Second, the available prostheses only provide limited choices in the contour footprint dimensions that cannot match various anatomic dimensions. Our findings are aligned with those of previous studies showing that the linear parameters of the cervical endplates vary among different ethnicities (Kim et al., 1976; Panjabi et al., 1976; Tan et al., 2004; Feng et al., 2017). In particular, the MLs in Caucasian cervical vertebrae are considerably larger than those reported in Asian subjects, as are the ML/AP values at the C3/4 and C4/5 segments. As the field of medicine continues to adopt 3D printing technologies, the use of 3D printing materials may provide a better quantitative understanding of anatomical implant design and help decrease postoperative complications.

The current study nevertheless had several limitations. One is the relatively small number of recruited subjects. Data from 130 Chinese subjects were collected retrospectively. Although geographical discrepancy was avoided in this analysis as much as possible, more patients should probably be included in future studies, which could minimize the problem with statistical bias. In addition, several other types and brands of cervical disc devices unapproved by the FDA for the treatment of cervical spondylosis and discogenic disease were not included in the present study. Lastly, we must acknowledge that the design characteristics of artificial disc was not only based on these anatomy parameters. There are several factors that provided challenges when trying to design a new generation disc. Although the aforementioned morphometric evaluation of cervical vertebrae is not a new subject, we will collect more data and extract more features to provide useful guidance and reference for the design of Chinese artificial discs with higher accuracy in future studies.

## Conclusion

Following data analysis, cervical measurements showed significant differences among different segments and sexes. There was a large discrepancy between the cervical anatomical data of Chinese individuals and the sizes of currently available prostheses. This study may provide useful guidance and a reference for the design of artificial discs for Chinese populations.

## Data Availability

The original contributions presented in the study are included in the article/supplementary material, further inquiries can be directed to the corresponding author.

## Abbreviations

CT, computed tomography; AP, anterior-posterior diameter; DH, middle disc height; ADH, anterior disc height; PDH, posterior disc height; ML, mediolateral diameter; ACDF, anterior cervical decompression and fusion; CDR, cervical disc replacement; PACS, picture archiving and communication system; HO, heterotopic ossification; FDA, Food and Drug Administration.
